# Statistical primer: sample size and power calculations—why, when and how?†

**DOI:** 10.1093/ejcts/ezy169

**Published:** 2018-05-10

**Authors:** Graeme L Hickey, Stuart W Grant, Joel Dunning, Matthias Siepe

**Affiliations:** 1 Department of Biostatistics, Institute of Translational Medicine, University of Liverpool, Liverpool, UK; 2 Department of Academic Surgery, University of Manchester, Manchester, UK; 3 Department of Cardiothoracic Surgery, James Cook University Hospital, Middlesbrough, UK; 4 Department of Cardiovascular Surgery, University Heart Centre, Freiburg, Germany

**Keywords:** Statistics, Sample size, Power, Clinical trials, Hypothesis testing

## Abstract

When designing a clinical study, a fundamental aspect is the sample size. In this article, we describe the rationale for sample size calculations, when it should be calculated and describe the components necessary to calculate it. For simple studies, standard formulae can be used; however, for more advanced studies, it is generally necessary to use specialized statistical software programs and consult a biostatistician. Sample size calculations for non-randomized studies are also discussed and two clinical examples are used for illustration.

## INTRODUCTION

It is essential that clinical studies are well-designed. One key aspect of study design is the sample size, which is the number of patients (or experiment subjects/samples) required to detect a clinically relevant treatment effect. Even for simple studies, there are several things to consider when calculating the required sample size. These include the type of study, type of outcome, variance of the outcome, the significance level and power of the test and the minimal clinically relevant difference. For more complex studies, there might be additional considerations that make the calculation more complex. As sample size calculations are frequently included in medical studies and are crucial to the interpretation of the study, a thorough understanding of the process underlying the calculation is necessary. In this article, we provide an overview of the rationale, methodology, implementation and reporting of sample size calculations specifically for cardiothoracic surgeons.

## METHODOLOGY

### Why and when?

The sample size calculation is generally required at the study design stage, before patient enrolment has begun [1]. There are several reasons for this [2]. Firstly, from a scientific perspective, testing too few might lead to failure to detect an important effect, whereas testing too many might lead to detecting a statistically significant yet clinically insignificant effect. Secondly, from an ethical viewpoint, testing too many subjects can lead to unnecessary harm or potentially unnecessary sacrifice in the case of animal studies. Conversely, testing too few is also unethical, as an underpowered study might not contribute to the evidence-based field of medicine. Thirdly, from an economical perspective, testing too many will lead to unnecessary costs and testing too few will be potentially wasteful if the trial is unable to address the scientific question of interest. For this reason, many funders and institutional review boards require an *a priori* sample size calculation, which is included in the study protocol. Adaptive trial designs, whereby prespecified modifications can be made to the trial after its inception, can potentially improve flexibility and efficiency [3].

### Components

There are four principal components required to calculate the sample size (Table 1). These components are specified via parameters. Working under a hypothesis testing framework, we assume a null hypothesis (*H*_0_) and an alternative hypothesis (*H*_1_). In practice, we do not know the ‘truth’, so we base our inferences on a statistical test applied to a random sample from the population. Two types of error can occur. The first is a Type I error, where the null hypothesis is true, but we incorrectly reject it. The second is a Type II error, where the null hypothesis is false, but we incorrectly fail to reject it. Specification of the Type I (denoted as α) and Type II (denoted as β but more commonly reported as the complement: the power =1-β) error rate parameters are required for the sample size calculation. Conventional choices are α=0.05 and 0.01 (corresponding to a significance level of 5% and 1%, respectively) and β=0.2 and 0.1 (corresponding to 80% and 90% power, respectively). However, there are situations in which these parameters might be increased or decreased, depending on the clinical context of the study [1].

**Table 1:** Primary components required for a sample size calculation

	What is it?	Specification
Type I error rate (α)	The probability of falsely rejecting *H*_0_ (false-positive rate)	Conventional choices are α=0.05 and 0.01
Power (1-β)	The probability of correctly rejecting *H*_0_ (true-positive rate), equivalent to 1 - the Type II error rate (β)	Conventional choices are β=0.20 and 0.10
Minimal clinically relevant difference	The smallest (biologically plausible) difference in the outcome that is clinically relevant	Input from the researcher(s) responsible for the study for the effect of scientific interest
Variance	Variability in the outcome (e.g. standard deviation for continuous outcomes)	Use existing clinical knowledge (e.g. other published articles) or a pilot study

The minimal clinically relevant difference is the smallest difference in outcome between the study groups, or effect size, that is of scientific interest to the investigator. For example, patients treated with a drug that lowers the mean systolic blood pressure by 7 mmHg compared to untreated hypertensive patients might be considered as clinically relevant (see Example 1). The statistician cannot decide this; it derives from clinical consideration. It is recognized that eliciting this minimal clinically relevant difference is difficult. A starting point is to ask: What results do you expect (or hope) to see? Typically, this question would be answered in terms of absolute effects, but relative effects can also be used. From the given response, the statistician and clinician can explore different scenarios about that choice. It is also useful to consider the individual benefit to the patient weighted against the potential inconvenience and adverse effects they might experience. The variance of the outcome is also required. This can generally be obtained from clinical knowledge; for example, if the clinician has historical similar data or if data have previously been published on the subject. In some cases, it is necessary to conduct a pilot study to gauge the variability of the outcome. Of course, in all cases, it must be considered whether the data used to determine the variance are reflective of the study sample in the planned study. If there are different eligibility criteria or outcome definitions, then this may not be the case.

### Calculations

A simple example of a sample size calculation is that of comparing two means for a continuous outcome. Assume that the null hypothesis is $H_{0}:\mu_{1}=\mu_{2}$ with an alternative hypothesis $H_{1}:\mu_{1}\neq \mu_{2}$, where $\mu_{1}$ is the true population mean in the control population, and $\mu_{2}$ the mean in the treated population. After collection of the data, a standard statistical test used to evaluate the hypothesis is the 2-tailed independent samples *t*-test. If the study investigators planned to have two groups of equal sample size n, then the following formula can be used, which is based on a normal approximation:

(1) $$n=\frac{2\sigma^{2}{\left(z_{1-\alpha /2}+z_{1-\beta }\right)}^{2}}{{\left(\mu_{1}-\mu_{2}\right)}^{2}},$$

where $\sigma^{2}$ is the common population variance for both populations, and $z_{1-\alpha /2}$ and $z_{1-\beta }$ are the 100(1-α/2) and $100\left(1-\beta \right)$ percentiles of the standard normal distribution, respectively. These values can be readily obtained using *z*-tables (Table 2) or statistical software with an example of this provided later in this article. Although other approximate formulae are sometimes used, a straightforward approximate formula [4] for comparing 2 proportions, $p_{1}$ and $p_{2}$, between groups is

(2) $$n=\frac{{\left\{z_{1-\alpha /2}\sqrt{2\bar{p}\left(1-\bar{p}\right)}+z_{1-\beta }\sqrt{p_{1}\left(1-p_{1}\right)+p_{2}\left(1-p_{2}\right)}\right\}}^{2}}{{\left(p_{1}-p_{2}\right)}^{2}},$$

where $\bar{p}=(p_{1}+p_{2})/2$.

**Table 2:** Conventional *z*-values for sample size calculations to use in Equations 1 and 2

α	$z_{1-\alpha /2}$
0.01	2.576
0.05	1.960
0.10	1.645
β	$z_{1-\beta }$
0.01	2.326
0.05	1.645
0.10	1.282
0.20	0.842

Based on these formulae, some immediate deductions can be made. First, the sample size is inversely proportional to square of the absolute effect size; hence, halving the effect size would require quadrupling the sample size. Second, reducing α or increasing the power (equivalent to reducing β) also increases the sample size required. Third, an increased variance leads to a larger necessary sample size. In the simple formula presented, the variance in each population was assumed to be identical. In practice, this may not hold, but adjustments are straightforward. It is clear, therefore, that slight changes in the factors that make up the sample size calculation (Table 1) can substantially alter the sample size. When there is doubt, it is generally advisable to err on the side of caution and choose the largest sample size of from the ensemble of scenarios. Although sample size formulae are frequently presented assuming 1:1 allocation between treatment and control groups, other allocation ratios can be accommodated using statistical software packages.

### Dropouts, missing data and other study deviations

Patient dropouts, non-adherence (or non-compliance) and missing data are, unfortunately, a common occurrence in clinical studies. It is, therefore, essential to consider the potential impact of this at the study design phase, as deviations may lead to a failure to achieve the intended aims of the trial. If missing data are anticipated completely at random, then the determined sample size can simply be inflated. That is, if the sample size required is n subjects per arm, and it is expected that up to x% patients will drop out, then the final sample size ($n^{*}$) can be inflated as $n^{*}=n/(1-\frac{x}{100})$. Attention must always be given to the reasons and mechanisms for missing data. Moreover, designing trials to minimize missing data is always the best approach [5]. Non-compliance, for example, due to patients crossing over to other treatment arms can also affect the power of a trial if not appropriately considered during the study design. The Arterial Revascularization Trial (ART) is an example of this (see Example 2). When different deviations can affect a study, all to varying degrees, simulation is the best approach to assess the potential impacts. In practice, these can be coded using standard programming languages (e.g. R), or specialist software can be utilized (Table 3).

**Table 3:** Software for sample size calculations

Software	Platform	URL^a^	Freely available?
**Stand-alone programs**			
G*Power	Windows and macOS	http://www.gpower.hhu.de	Yes
PS	Windows	http://biostat.mc.vanderbilt.edu/wiki/Main/PowerSampleSize	Yes
PASS	Windows	https://www.ncss.com/software/pass	No
nQuery	Windows	https://www.statsols.com/nquery-sample-size-and-power-calculation-for-successful-clinical-trials	No
**R^b^ packages**			
pwr	Windows, macOS and Linux	https://cran.r-project.org/web/packages/pwr	Yes
TrialSize	Windows, macOS and Linux	https://cran.r-project.org/web/packages/TrialSize	Yes
PowerUpR^c^	Windows, macOS and Linux	https://cran.r-project.org/web/packages/PowerUpR	Yes
powerSurvEpi	Windows, macOS and Linux	https://CRAN.R-project.org/package=powerSurvEpi	Yes
**SAS**			
PROC POWER	Windows and Linux	https://support.sas.com/documentation/cdl/en/statug/63033/HTML/default/viewer.htm#power_toc.htm	No
**SPSS**			
SamplePower	Windows	https://www-01.ibm.com/marketing/iwm/iwmdocs/tnd/data/web/en_US/trialprograms/U741655I36057W80.html	No
**Stata**			
power	Windows, macOS and Linux	https://www.stata.com/features/power-and-sample-size/	No
**Microsoft Excel**			
PowerUp^c^		http://www.causalevaluation.org/power-analysis.html	Yes^d^
**Specialist simulation software**			
IcebergSim	Windows	http://icebergsim.software.informer.com/versions/	Yes
FACTS	Windows	https://www.berryconsultants.com/software/	No
Clinical trial simulation	Windows and Linux	http://www.biopharmnet.com/innovation/trial_simulation/cts1.php	Yes^e^

a URLs are correct as of 11 April 2018.

b R also has several base functions that enable power calculations to be made; e.g. power.t.test(), power.prop.test() and power.anova.test().

c Specialized package for the case of cluster (multilevel) trials.

d Requires Microsoft Excel to be installed.

e Requires SAS to be installed.

### Estimation

When interest lies in *estimation* of a quantity, rather than *hypothesis testing*, then sample size calculations can be reframed to control the expected width of the confidence interval. For example, a surgeon might want to estimate—with a certain accuracy—the proportion, p, of patients undergoing cardiac surgery who would fail a preoperative cardiopulmonary exercise stress test. As cardiopulmonary exercise stress testing is expensive and time consuming to perform, the surgeon wants to estimate this proportion with a margin of error <5%. The estimated proportion will be $\hat{p}=\left\{number\;of\;patients\;that\;\text{failed}\right\}\}/n$, where n is the number of patients required to be tested. Standard mathematical approximations give a 95% confidence interval as

$$\hat{p}\pm z_{0.975}\sqrt{\frac{\hat{p}(1-\hat{p})}{n}}.$$

The term after the plus–minus sign is the margin of error, with the square root term denoting the standard error (SE). The surgeon does not know the value of $\hat{p}$ yet and, therefore, does not know the SE, but a ‘worst case’ scenario can be mathematically determined based on $\hat{p}=0.5$. The margin of error is then approximately 1/√n, which the surgeon in the example above specified must be <5%; hence, it is required that n is ≥400. If the surgeon has an estimate of the prevalence, then this sample size can potentially be reduced by refining the SE estimate.

### Software

There are a large number of software programmes available for performing sample size calculations (see Table 3 for a non-exhaustive list of available programmes) including stand-alone programmes, which might be preferable for those unfamiliar with general statistical software programmes. The capabilities of each vary from basic to complex calculations, but common trial designs are integrated into most. There are many online sample calculators and even some smartphone apps that can perform sample size calculations. Although such calculators can be useful, it should be noted that they are unlikely to have been validated, and it is generally advisable to use a validated software programme. Some potentially useful calculators can be found at http://homepage.stat.uiowa.edu/∼rlenth/Power/, ‘Power and Sample Size’ (http://powerandsamplesize.com) and OpenEpi (http://www.openepi.com) [all accessed 11 April 2018]. There are also several specialist programs available to conduct simulation analyses to explore deviations (e.g. non-compliance, dropouts and adaptive designs).

### Reporting

It is essential that the details of the sample size calculation are reported in full. This demonstrates that the study has been well designed and establishes what the primary outcome is on which the calculation is based. It also enables reproducibility of the study. Reporting the calculation requires reporting of all parameters and assumptions used. In some cases, sample size formulae depend only on the standardized difference. However, it is preferable to report the individual components (expected effect size in each treatment group and variability). Details of inflation factors (e.g. due to expected dropouts) should also be reported. It is worthwhile noting that reporting of sample sizes is a requirement of the CONSORT Statement checklist [6]. The STROBE Statement checklist also asks for details on how the sample size was arrived at to be provided [7].

### Non-randomized studies

Sample size formulae are generally presented for randomized clinical trials. In some cases, randomization is not feasible due to physical constraints, ethical issues or resource limitations. This leads to observational studies, whereby due to the presence of confounding variables, it is not appropriate to apply univariable comparison tests due to potential bias. In addition, there is potentially increased measurement error and unknown enrolment protocols. Consequently, sample size calculations in such studies are more complex. In some cases, analyses are based on large clinical (or administrative) registries. These can be very large, meaning that there is little concern about power. However, it is then important to consider whether the adjusted effect size is ‘clinically’ significant, regardless of ‘statistical’ significance. Another issue frequently encountered with non-randomized studies, particularly observational data studies, is the presence of missing data. In some cases, this can be substantial. Complete case analysis (i.e. excluding patients with any missing data) would likely lead to bias and larger SEs, whereas imputation techniques would need to account for the additional uncertainty from the imputed data [8].

A standard approach for adjusting for confounding is multivariable regression. In such models, we are typically interested in a single covariate (e.g. a binary treatment effect) and will include other covariates (i.e. the confounders) in the regression model. For multivariable linear regression, one approach is to apply a correction factor to the approximate formula (cf. Equation 1) [9]. Formulae for other regression models are also available, e.g. the Cox proportional hazards model [10] and the logistic [11] regression model. In the case of the former, it is a sample size formula for the number of ‘events’ required rather than the number of ‘subjects’. An alternative approach frequently used in non-randomized cardiothoracic surgery research studies is propensity score analysis using matching, covariance adjustment, inverse probability treatment weights or stratification [12]. Sample size calculations need to take account of the method used. For example, if 1-to-1 propensity score matching is used, then a sample size calculated assuming randomization must be inflated to account for patients who will not be matched based on the ratio of controls to treated patients and the degree of overlap of propensity score distributions [13]. In either case, the precision of the estimated treatment effect following adequate adjustment should be appropriately reported and interpreted.

### When the sample size is not achievable

In some cases, achieving the determined sample size will not be feasible due to external factors; for example, time or resource limitations. This frequently happens in rare diseases, e.g. in congenital cardiac surgery. The immediate question is how to proceed in such a circumstance. One option is to inverse the problem and calculate the power that can be attained from the maximum permissible sample size. This power can then be evaluated against the study objectives. If the power is insufficient, then it might be used to gain additional funding to recruit further subjects.

If recruiting more subjects is not feasible, then other options include changing the primary outcome (e.g. using a composite outcome that increases the number of events) [14], pooling resources and sample populations with other centres and exploring means of reducing the variability (e.g. by limiting the scope of the patient population). Perhaps, the least desirable option is to simply not proceed with completing the study. In this case, data from the study might still be published, as there is a body of methodologists that consider underpowered trials to be acceptable [1, 15]. The rationale for considering underpowered studies is that they can potentially be combined with other small studies in a meta-analysis framework. There is also a notion that some knowledge is better than no knowledge. Caveats exist in pursuing such an approach, which include the requirement of absolute transparency, and the rigorous minimization of potential sources of bias (e.g. due to inadequate randomization, blinding or retention) [1].

## EXAMPLES

### Example 1: watermelon treatment for hypertension

Following a pilot study by Figueroa *et al.* [16], a fictitious team of investigators want to design a study to test the effect of watermelon extract on systolic blood pressure among hypertensive patients. The investigators hypothesized that the L-citrulline present in watermelon, which naturally converts to L-arginine, will increase the production of endothelial nitric oxide and thus have a vasodilatory effect. Conventional choices of the Type I error rate (α=0.05) and power (1-β=0.80) are proposed, yielding *z*-values of 1.96 and 0.84 (see Table 2), respectively, in Equation 1. The investigators plan to compare the systolic blood pressure at 6 weeks between placebo and daily watermelon treatment groups using an independent *t*-test. Based on previous knowledge, it was assumed that mean baseline systolic blood pressure would be 140 mmHg in the control group, and the researchers believed that a biologically plausible reduction of 5% systolic blood pressure (7 mmHg) in the treatment group would be of scientific interest. Assuming a common standard deviation of 10 mmHg in both treatment arms, the necessary sample size (Equation 1) would be 33 subjects per treatment arm. Using a more accurate method (e.g. using a software package such as G*Power 3.1; Table 3), we would be able to determine that a sample size of 34 subjects per group would be required, confirming the approximate formula (Equation 1) as being sufficiently accurate for practical application.

### Example 2: Arterial Revascularization Trial (ART)

The ART is a randomized controlled clinical trial initiated in 2004 with the primary objective to compare 10-year survival associated with using bilateral internal thoracic arteries versus the use of a single internal thoracic artery graft for coronary artery bypass surgery [17]. Following a systematic review, the investigators expected mortality of 25% in the single internal thoracic artery arm and 20% in the bilateral internal thoracic artery arm, conferring an absolute effect size of 5%. After specifying α=0.05 and β=0.10 (for 90% power), the authors estimated that a total sample size of 2928 patients (*n *=* *1464 per treatment arm) would be required. This can be calculated by the sample size formula proposed by Freedman for comparing survival curves using the log-rank test [18]. Here, we used the ‘ssizeCT.default’ function in the R package powerSurvEpi (Table 3; in this case, the sample size calculated was *n* = 1463 per arm). The authors subsequently rounded this up to 3000 patients (*n *=* *1500 per treatment arm), with 3102 patients actually randomized. A limitation of the ART study is that there was substantial non-compliance (16.4% randomized to the bilateral internal thoracic artery did not receive this treatment, versus 3.9% randomized to single internal thoracic artery group who were non-compliant with treatment allocation). In addition, several patients were lost to follow-up, which will affect the overall power achieved [19].

## DISCUSSION

The sample size calculation is a crucial element of study design. However, it is only one element of a well-designed protocol. For basic study designs and outcomes, several sample size formulae exist in medical statistics textbooks. For more advanced study designs or situations, there exist specialized textbooks [20] and accessible software programs (Table 3). In addition, for the most complex cases, experienced statisticians can use simulation methods to determine the sample size [21]. Nonetheless, we would generally advise the involvement of a statistician in all but the most basic trial designs. A fundamental requirement after the sample size calculation has been performed is the clear and transparent reporting of it [6]. A review of 6 high-impact journals found that 5% did not report any details and 43% did not report all the parameters necessary to reproduce the calculations [22].

There has been a perception that sample sizes of randomized controlled trials (RCT) in specialty fields such as cardiovascular medicine have increased over the years. The median sample size used in *Circulation* and the *European Heart Journal* in 1990 was 99 and 90, which rose to 630 and 935 in 2010, respectively [23]. One proposed explanation is that larger treatment effects have already been identified in historical studies, leaving only small effects to be discovered through more contemporary studies. Commensurate with increasing sample sizes are increased costs, study periods and resources. It is, therefore, necessary to not only consider sample size but also alternative study designs that can reduce these burdens. For example, (Bayesian) adaptive trials are one approach, whereby parameters specified in the trial protocol can be modified as observations are accumulated [24]. These adaptations must be specified in advance according to predefined rules and might include interim analyses with the aim of possibly stopping the trial early (e.g. due to success or futility), sample size re-estimation or changes to the randomization procedure.

Sample size calculations are sensitive to parameter choices and, hence, errors. Exploring a range of scenarios with regard to the sample size calculation can provide insight into the potential practical consequences. Sample size calculations should always be performed *a priori* since ‘*post hoc* power calculations’ have no value once the study has concluded [1]. If the sample size was not calculated *a priori*, then this should be acknowledged, and the uncertainty in the treatment effect demonstrated should be represented via a confidence interval.

## Funding

GLH was supported by the Medical Research Council (MRC) [MR/M013227/1 awarded to Dr Ruwanthi Kolamunnage-Dona (University of Liverpool)].

**Conflicts of interest:** none declared.
